# Association of low-carbohydrate diet score with overweight, obesity and cardiovascular disease risk factors: a cross-sectional study in Iranian women

**DOI:** 10.15171/jcvtr.2019.36

**Published:** 2019-08-26

**Authors:** Soudeh Jafari-Maram, Elnaz Daneshzad, Neil R. Brett, Nick Bellissimo, Leila Azadbakht

**Affiliations:** ^1^Department of Community Nutrition, School of Nutritional Sciences and Dietetics, Tehran University of Medical Sciences, Tehran, Iran; ^2^School of Nutrition, Ryerson University, Toronto, Ontario, Canada; ^3^Diabetes Research Center, Endocrinology and Metabolism Clinical Sciences Institute, Tehran University of Medical Sciences, Tehran, Iran

**Keywords:** Low-Carbohydrate Diet, Overweight, Obesity, Cardiovascular Disease, Women

## Abstract

***Introduction: *** This study aimed to determine the association of low-carbohydrate-diet score with overweight, obesity and cardiovascular risk factors among Iranian women.

***Methods:*** In healthy Iranian women 20-50 years, demographics, anthropometrics, physical activity, blood pressure, fasting blood glucose, blood lipids, and dietary intake (using a validated food frequency questionnaire) were assessed. Participants were divided into deciles of macronutrient intakes. Women in the lowest decile of carbohydrate intake received a score of 9 and women in the highest decile received a score of 0. For protein and fat intakes, women in the lowest decile received a score of 0 for that macronutrient and those in the highest decile received the score of 9. Macronutrient scores were summed to create the low-carbohydrate diet score and women were grouped into tertiles based on these scores. Continuous and qualitative variables were compared among the low-carbohydrate-diet score by one-way ANOVA and chi-square test, respectively. Logistic regression was used to determine the association of low-carbohydrate-diet score and cardiovascular risk factors.

***Results:*** A total of 209 women were included in the study. Socioeconomic status significantly increased from tertile 1 to 3 of the low-carbohydrate diet score (*P * = 0.02). Total dietary glycemic index (GI) significantly differed among tertiles (tertile 1 GI: 63.1 ±0.50, tertile 2 GI: 61.9 ± 0.5, tertile 3 GI: 59.5 ± 0.5; *P * < 0.001). The odds ratios for overweight, obesity and cardiovascular risk factors were not significantly different among the tertiles of low-carbohydrate diet score.

***Conclusion:*** In Iranian women, diets lower in carbohydrate and higher in protein and fat were not associated with overweight, obesity and cardiovascular risk factors.

## Introduction

In 2014, more than 1.9 billion adults were overweight and over 600 million were obese.^1^ Women of reproductive age are one of the groups at high risk for obesity.^2^ Obesity and overweight are linked to an increased risk of chronic disease.^3^ In Iran, the combined prevalence of overweight and obesity may be as high as 76% in some regions.^4^ The prevalence of obesity in Iran is greater in women than men^2,5,6^ and metabolic syndrome and hyper-triglyceridemia, which are clinical sequelae of obesity, are more prevalent in Iranian women.^7^ Further, hyper-triglyceridemic waist-phenotype, which is linked with high carbohydrate consumption, is a determinant of atherogenesis and mortality from cardiovascular disease in women.^8^ In addition to hypertriglyceridemia, the most prevalent components of dyslipidemia among Iranian women is low high-density lipoprotein (HDL),^9^ which may also relate to high carbohydrate intake, particularly from refined sources.

As the main source of energy in Iran is carbohydrates,^10^ it is important to further understand how carbohydrate intake relates to the prevalence of overweight, obesity and risk factors for cardiovascular disease. A low–carbohydrate (low-CHO) diet is defined as a dietary pattern that consists of <45% of energy from carbohydrates.^11^ Diets that limit carbohydrate consumption have become a popular strategy for weight loss.^12^ However, it is unclear whether low-CHO diets improve biomarkers of health or cardiovascular disease, as results are mixed from the Nurses Health Study,^13^ a recent meta-analysis^14^ and other work.^15,16^ When evaluating carbohydrate intake, scoring of macronutrient intakes has become more frequently used as it allows a researcher to examine the relationship between different levels of intake and the risk of illness. Each macronutrient may have various effects on metabolic risk factors,^10,13,15-17^ and these scores consider the proportion of all 3 macronutrients from total energy intake. There is no previous work investigating the relationship between low-CHO diet scores with overweight, obesity and cardiovascular risk factors in Iranian women. Therefore, the objective of this study was to determine the relationship between low-CHO diet score and overweight, obesity and cardiovascular risk factors among a group of Iranian women.

## Materials and Methods

The STROBE statement was followed for designing and reporting this study (Supplementary file 1). For this cross-sectional study, the sample size was calculated as follows: n= [(z_1_ -α⁄2)^2^ ×s^2^] ⁄d^2^. According to previous studies, body mass index (BMI) was considered as the main basis for calculating sample size (BMI: 26.5 ± 3.5; α: 0.05; d: 2% × 26.5).^18^ Considering the multi-stage cluster sampling method, the design effect was considered 1.25. Eventually, 209 women entered the study.

Multistage cluster sampling was conducted from August 2016 to March 2017. Women interested in participating were referred to health centers in Tehran University of Medical Sciences. In the first stage of sampling, all of the health centers in each of the urban geographic regions (North, South, East, West, and Central) were identified and an equal number of health centers from each region were randomly selected. An equal number of women were then randomly sampled from each region. Written informed consent was given by all participants in the study. Inclusion criteria included women 20-50 years, being Iranian, nonimmigrant, not being pregnant, not lactating, pre-menopausal and lack of chronic diseases such as diabetes, thyroid disease, cardiovascular disease, cancer, liver disease, and kidney disease. Persons who underreported or overreported energy intake (less than 800 calories or more than 4200 calories) were excluded from the study.^19^

### Dietary intake assessment

The nutritional status of women was evaluated by a validated and reliable Iranian food frequency questionnaire with 168 items.^9^ In this questionnaire, standard sizes for every food item was designed according to the method of Willett et al.^20^ The amount of each food item eaten was then converted to grams per day. The subjects were asked to indicate their frequency of intake of each food item according to the amount of food consumed per day, week, or month during the previous year.

The total dietary glycemic index (GI) was estimated by using the following formula: ∑ (GI_a_ × available carbohydrate_a_)/total available carbohydrate. Additionally, GI for each Iranian food was derived from national references and measurements,^21^ while the GI of other foods was derived from international references.^22^ GI values of unavailable foods, such as Gaz was estimated based on chemically and physically similar foods.^23^ Glucose was considered as the reference for all derived GI values.

The low-CHO diet score was calculated for each participant. Participants were divided into decile of carbohydrate, protein and fat intake as a percentage of energy intakes. For carbohydrate, women in the lowest decile of carbohydrate intake received a score of 9 and women in the highest decile received a score of 0. For protein and fat intakes, women in the lowest decile received a score of 0 for that macronutrient and those in the highest decile received the score of 9. Then to create the low-CHO diet score, macronutrient scores were summed, with a range of 0 to 27. A score of 0 indicates the highest carbohydrate intake and the lowest fat and protein intake and a score of 27 indicates the lowest carbohydrate intake and the highest protein and fat intake.^10^ Participants then grouped into tertiles of low-CHO diet scores (tertile 1: <10, tertile 2: 10 – 16, tertile 3: ≥ 17).

### Demographics, anthropometrics and physical activity assessment

A questionnaire was used to determine the age, gender, socioeconomic status, and history of diseases, family history of diseases, as well as the use of drugs that affect blood lipids, blood pressure, blood glucose, thyroid drugs, corticosteroids, cardiovascular drugs, and hormonal contraceptives in the last 3 months. Weight, height and waist circumference were measured for each woman. Measurements were performed according to the International Standards for Anthropometric Assessment.^24^ Height and waist circumference was recorded to the nearest 0.1 cm and weight to the nearest 0.1 kg. Anthropometric measurements were carried out without shoes and wearing light clothing. Waist circumference was measured at the narrowest area of the waist. Normal waist circumference was defined as ≤88 cm for women.^25^ BMI was also calculated by dividing weight in kilograms by height in meters squared. Overweight was defined as BMI 25.0 to 29.9 kg/m^2^ and obesity as BMI ≥30.0 kg/m^2^. The International Physical Activity Questionnaire (IPAQ) was used to assess physical activity over the previous 7 days.^26^ Activity was then converted to metabolic equivalent (MET).h per day. To calculate this, type and hours of all activities, such as running, walking, sitting activities, sleeping hours, etc were recorded and then the number of hours per day of activity was multiplied by the coefficient for each activity.

### Blood pressure assessment and biochemical measures

Before blood pressure was measured, participants were asked about consumption of coffee and tea and recent physical activity. Blood pressure was measured using a standard mercury sphygmomanometer, with appropriate cuffs, after the subject had been at sitting at rest for 15 minutes. The mean of two measurements was calculated for each individual. High blood pressure was defined as systolic blood pressure ≥ 130 mm Hg and/or diastolic blood pressure ≥85 mm Hg.^27^

To measure blood glucose and serum lipids, blood samples were collected after 10-12 hours of fasting. To measure blood lipids (including total cholesterol, triglycerides, Low-density lipoprotein [LDL] cholesterol, and HDL cholesterol), a 10 mL venous blood sample was obtained and then centrifuged for 10 min. Total cholesterol, triglyceride, LDL-cholesterol and HDL-cholesterol concentrations were measured by commercial kits (Pars Azmoon, Tehran, Iran).^10^ Elevated TG levels were defined as fasting serum TG ≥1.69 mmol/L. Low serum HDL cholesterol was defined as HDL < 1.29 mmol/L. High serum total cholesterol level was defined as total cholesterol >5.18 mmol/L. High serum LDL cholesterol was defined as LDL cholesterol >2.59 mmol/L. Fasting glucose concentration was measured using the glucose oxidase technique.^28^ Abnormal fasting blood glucose concentration was defined as ≥5.55 mmol/L.

### Statistics

Statistical analysis was performed with the Statistical Package for Social Sciences (version 16; SPSS Inc, Chicago, IL, USA). P < 0.05 was considered statistically significant. Participants were categorized according to tertiles of low-CHO diet score (tertile 1: < 10, tertile 2: 10 – 16, tertile 3: ≥ 17). Continuous variables were compared among the low-CHO diet score by one-way ANOVA. In the case of qualitative variables, the distribution of individuals among the tertiles was calculated using chi-square test. Also, the mean dietary intakes of the subjects were compared among tertiles using ANOVA adjusted for energy intake. Logistic regression was used to determine the relationship between low-carbohydrate-diet score with overweight, obesity and cardiovascular risk factors. In the first model, only age was adjusted for, whilst the second model was adjusted for age, smoking, physical activity, use of oral estrogen as well as family history of diabetes and stroke. Model 3 also included energy intake as a covariate. In all models, the first tertile was considered as the reference group.

## Results

General characteristics of individuals, overweight, obesity, and any of the cardiovascular risk factors among tertiles of the low-carbohydrate- diet score are shown in Table 1. As shown, the mean age (*P *= 0.11), BMI (*P *= 0.72), waist circumference (*P *= 0.96), physical activity (*P *= 0.17), family history of diabetes (*P *= 0.33), family history of heart disease (*P *= 0.16) and cardiovascular risk factors (*P *= 0.69, 0.54, 0.43, 0.78, 0.26, 0.98, 0.92 for fasting blood glucose, triglyceride, total cholesterol, HDL-cholesterol, LDL-cholesterol, systolic blood pressure and diastolic blood pressure, respectively) were not significantly different among tertiles of the low-carbohydrate diet score. Regarding the socioeconomic level of the family, there was a significant difference among the tertiles of low-carbohydrate diet score (*P *= 0.02).

**Table 1:** Characteristics of Iranian women by tertiles of the low-carbohydrate diet score^a^

	**T1**		**T2**		**T3**		***P*** **value** ^b^
	**Mean**	**SD**	**Mean**	**SD**	**Mean**	**SD**	
Cut points of tertiles	≤ 9		10 – 16		≥ 17		
Participants n	69		64		76		
Age (y)	31.54	9.30	28.78	8.54	29	7.65	0.11
BMI (kg/m^2^)	24.43	4.23	23.93	4.12	23.95	4.02	0.72
WC (cm)	82.94	10.27	82.87	11.04	82.50	10.81	0.96
Physical activity (MET-h/wk.)	26.74	3.31	27.50	3.95	27.99	4.57	0.17
Family history of diabetes (%)	27.5		18.8		28.9		0.33
Family history of heart disease (%)	24.6		12.5		15.8		0.16
Socioeconomic status of the family^c^							0.02
Weak (%)	44.9		32.8		22.4		
Average (%)	34.8		29.7		40.8		
Strong (%)	20.3		37.5		36.8		
Fasting blood glucose (mmol/L)	4.92	1.42	4.95	0.75	4.81	0.75	0.69
Model I^d^	4.90	1.00	4.96	1.00	4.82	1.00	0.70
Triglyceride (mmol/L)	1.16	0.68	1.20	0.84	1.07	0.57	0.54
Model I	1.15	0.66	1.21	0.65	1.08	0.65	0.53
Total cholesterol (mmol/L)	4.50	1.03	4.62	0.86	4.41	0.93	0.43
Model I	4.48	0.93	4.63	0.93	4.42	0.93	0.41
HDL cholesterol (mmol/L)	1.22	0.24	1.25	0.26	1.22	0.26	0.78
Model I	1.22	0.25	1.24	0.25	1.22	0.25	0.83
LDL cholesterol (mmol/L)	2.03	0.50	2.08	0.49	1.94	0.51	0.26
Model I	2.03	0.50	2.08	0.50	1.95	0.50	0.26
Systolic blood pressure (mm Hg)	113.9	11.0	113.9	9.80	113.6	9.70	0.98
Model I	113.7	9.10	114.0	9.60	113.7	9.50	0.98
Diastolic blood pressure (mm Hg)	78.0	7.20	77.9	6.80	77.60	6.20	0.92
Model I	77.9	6.60	78.0	6.40	77.60	6.10	0.94

BMI, body mass index; FPG, fasting plasma glucose; HDL, high-density lipoprotein; TG, triacylglycerol; WC, waist circumference; LDL, low-density lipoprotein ^a^ Values are means ± SD; ^b^*P* values result from ANOVA for quantitative variables and chi-square test for qualitative variables; ^c^The socioeconomic status of participants was estimated with a scoring method based on responses to questions which related to their education level, housing estate status, housing area, personal car, family income, and the frequency of recreational travel. All scores were summed and the overall score was categorized in tertile; ^d^Controlled for BMI.

Dietary intakes of women across tertiles of the low carbohydrate- diet score are shown in Table 2. According to this table, women in the highest tertile of this score had a higher consumption of meat, various types of fats including cholesterol, saturated fatty acids (SFA), monounsaturated fatty acid (MUFA), and poliunsaturated fatty acids (PUFA) (*P *< 0.001) and dairy products (*P *= 0.02). The consumption of fruits inversely related to tertiles (*P *= 0.01). Consumption of vegetables was not significantly different among the tertiles of the low-carbohydrate diet score (*P *= 0.53). Further, total dietary GI significantly differed among tertiles (tertile 1 GI: 63.1 ± 0.50, tertile 2 GI: 61.9 ± 0.5, tertile 3 GI: 59.5 ± 0.5; *P *< 0.001). Odds ratios for overweight, obesity and cardiovascular risk factors among the tertiles of the low-CHO diet scores are presented in Table 3. As shown in this table, before and after confounders adjustment (model 1: age, model 2: smoking, physical activity, socioeconomic status, estrogen consumption and family history of diabetes and stroke, model 3: confounders in model 2 and energy intake) the odds ratio for overweight, obesity, abdominal obesity and any of the cardiovascular risk factors was not significant among the tertiles of low-CHO diet tertiles.

**Table 2:** Dietary intakes of Iranian women by tertiles of the low-carbohydrate diet score^a^

	**T1**		**T2**		**T3**		***P*** **value** ^c^
	**Mean**	**SD**	**Mean**	**SD**	**Mean**	**SD**
Cut points of tertiles	≤ 9		10 – 16		≥ 17		
Participants, n	69		64		76		
Total energy (kcal)	2445	700	2635	819	2524	763	0.34
Carbohydrate (% energy)	67 0.03		60 0.02		53 0.04		<0.001
Protein (% energy)	13 0.01		13 0.02		16 0.03		<0.001
Fat (% energy)	21 0.03		27 0.03		32 0.04		<0.001
Cholesterol (mg)							
Crude	167	76	245	140.0	284.2	140.8	<0.001
Model I^b^	174	105	236	104.7	284.9	104.4	<0.001
SFA (g)							
Crude	18.3	6.9	26.0	9.9	28.8	11.1	<0.001
Model I	19.2	5.6	25.0	5.5	28.8	5.5	<0.001
MUFA (g)							
Crude	18.3	7.4	25.1	8.9	28.8	10.3	<0.001
Model I	19.2	4.4	24.1	4.4	28.8	4.4	<0.001
PUFA (g)							
Crude	14.8	7.1	19.8	7.8	22.6	9.0	<0.001
Model I	15.4	6.1	19.0	6.1	22.6	6.1	<0.001
Whole grains^d^(g)							
Crude	146	108	121	72	88	65	<0.001
Model I	149	79	117	80	88	79	<0.001
Refined grains^e^ (g)							
Crude	420	232	398	191	291	164	<0.001
Model I	431	174	385	176	293	174	<0.001
Vegetables^f^ (g)							
Crude	366	240	388	241	411	243	0.53
Model I	377	223	374	223	412	222	0.51
Meat^g^ (g)							
Crude	98	50	142	63	187	92	<0.001
Model I	102	64	137	64	188	63	<0.001
Dairy^h^ ( g )							
Crude	404	239	462	273	535	350	0.02
Model I	423	248	438	250	538	247	0.01
Fruit^i^ (g)							
Crude	392	294	365	265	277	181	0.01
Model I	403	230	352	230	278	229	0.005
Fats^j^(g)							
Crude	76	47	108	56	106	49	<0.001
Model I	80	39	103	39	106	39	<0.001
Glycemic index^k^	63.1	0.5	61.9	0.5	59.5	0.5	<0.001

BMI, body mass index; FPG, fasting plasma glucose; HDL, high-density lipoprotein; TG, triacylglycerol; WC, waist circumference; LDL, low-density lipoprotein ^a^ Values are means ± SD; ^b^ Adjusted for energy intake; ^c^*P* values result from the ANOVA test for the crude model and ANCOVA test for the adjusted model; ^d^ Includes whole-grain bread, dietary bread, cornflakes, popcorn, wheat flour; ^e^ Includes white bread, noodles, pasta, rice, and biscuits; ^f^ Includes lettuce, tomatoes, mixed vegetables, cucumbers, pumpkins, eggplant, celery, green peas, green beans, carrots, garlic, onions, cabbage, sweet pepper, spinach, turnips, and mushrooms; ^g^Includes lentils, beans, peas, beans, leeks, eggs, beef or calves, lamb, chicken, fish, tuna fish, hamburger, sausages and lamb gut; ^H^ Includes milk, chocolate milk, cheese, cream, yogurt, cream cheese, dough, whey, and ice cream; ^I^ Includes melon, watermelon, pear, apricot, cherry, apple, peach, nectarine, green tomato, fresh fig, dried figs, grapes, kiwi, grapefruits, oranges, persimmons, mandarin, pomegranate, palm, plums, cherries, berries lemons, lemons, sour Lime, fresh pineapple, pineapple canned, raisins, fresh berries, dried berries, peach sheets, apricot plum, compote fruits, green olive; ^J^ Includes varieties of oils, mayonnaise, peanuts, almonds, walnuts, pistachios, hazelnuts, seeds, butter, cream, sweets, chocolate, caramel cream, chips, pizza, halvah, and donuts; ^K^ values are mean ± SD and adjusted for energy intake

**Table 3:** Odds ratio and 95% CI for overweight, obesity and cardiovascular risk factors among the tertiles of the low-carbohydrate diet score in Iranian women

	**T1**	**T2**	**T3**	***P*** **value** ^a^
	**OR (95% CI)**	**OR (95% CI)**	**OR (95% CI)**	
Cut points of tertiles	≤ 9	10–16	≥ 17	
Participants (n)	69	64	76	
**Overweight (n= 55)**				
Crude	1	0.68 ( 0.31–1.50 )	0.93 ( 0.45–1.92 )	0.87
Model I^b^	1	0.78 ( 0.34–1.75 )	1.07 ( 0.50–2.25 )	0.84
Model II^c^	1	0.76 ( 0.33–1.78 )	1.11 ( 0.50–2.47 )	0.77
Model III^d^	1	0.68 ( 0.31–1.51 )	0.93 ( 0.45–1.92 )	0.87
**Obesity (n= 22)**				
Crude	1	0.72 ( 0.25–2.03 )	0.41 ( 0.13–1.28 )	0.12
Model I	1	0.92 ( 0.31–2.75 )	0.53 ( 0.16–1.71 )	0.30
Model II	1	1.22 ( 0.39–3.81 )	0.80 ( 0.23–2.84 )	0.81
Model III	1	0.66 ( 0.23–1.90 )	0.39 ( 0.12–1.24 )	0.10
**Abdominal obesity (n= 63)**				
Crude	1	0.74 ( 0.35–1.53 )	0.58 ( 0.28–1.19 )	0.14
Model I	1	0.93 ( 0.42–2.06 )	0.71 ( 0.33–1.53 )	0.38
Model II	1	1.03 ( 0.45–2.37 )	0.83 ( 0.36–1.91 )	0.62
Model III	1	0.71 ( 0.34–1.49 )	0.57 ( 0.28–1.18 )	0.13
**High FPG (n= 21)**				
Crude	1	0.93 ( 0.31–2.74 )	0.65 ( 0.21–1.98 )	0.45
Model I	1	1.76 ( 0.46–6.68 )	1.32 ( 0.35–5.00 )	0.64
Model II	1	1.83 ( 0.41–8.22 )	1.25 ( 0.28–5.61 )	0.64
Model III	1	0.93 ( 0.31–2.75 )	0.65 ( 0.21–1.98 )	0.45
**High serum TG concentration (n= 30)**				
Crude	1	1.36 ( 0.54–3.41 )	0.69 ( 0.25–1.87 )	0.47
Model I	1	2.03 ( 0.73–5.63 )	0.97 ( 0.33–2.84 )	0.98
Model II	1	2.17 ( 0.74–6.40 )	1.01 ( 0.30–3.33 )	0.81
Model III	1	1.28 ( 0.50–3.25 )	0.67 ( 0.24–1.83 )	0.44
**Low serum HDL concentration (n= 132)**				
Crude	1	0.93 ( 0.46–1.86 )	1.67 ( 0.84–3.34 )	0.14
Model I	1	0.98 ( 0.49–1.98 )	1.76 ( 0.87–3.54 )	0.10
Model II	1	0.94 ( 0.44–2.02 )	1.82 ( 0.83–3.96 )	0.15
Model III	1	0.93 ( 0.46–1.87 )	1.67 ( 0.84–3.34 )	0.14
**High serum total cholesterol concentration (n= 49)**				
Crude	1	1.40 ( 0.63–3.10 )	0.96 ( 0.43–2.12 )	0.90
Model I	1	1.80 ( 0.77–4.16 )	1.19 ( 0.51–2.74 )	0.71
Model II	1	1.84 ( 0.77–4.41 )	1.21 ( 0.49–2.97 )	0.71
Model III	1	1.40 ( 0.63–3.09 )	0.95 ( 0.43–2.12 )	0.89
**High serum LDL concentration (n= 25)**				
Crude	1	0.64 ( 0.23–1.78 )	0.53 ( 0.19–1.46 )	0.21
Model I	1	0.75 ( 0.26–2.12 )	0.62 ( 0.22–1.74 )	0.36
Model II	1	0.62 ( 0.20–1.95 )	0.53 ( 0.17–1.68 )	0.32
Model III	1	0.64 ( 0.23–1.79 )	0.53 ( 0.19–1.46 )	0.21
**High systolic blood pressure (n= 13)**				
Crude	1	2.27 ( 0.54–9.51 )	1.22 ( 0.26–5.66 )	0.84
Model I	1	3.05 ( 0.69–13.48 )	1.65 ( 0.34–8.02 )	0.55
Model II	1	3.60 ( 0.62–20.71 )	1.05 ( 0.14–7.79 )	0.79
Model III	1	1.97 ( 0.46–8.43 )	1.14 ( 0.24–5.36 )	0.92
**High diastolic blood pressure (n= 26)**				
Crude	1	1.41 ( 0.52–3.83 )	0.89 ( 0.31–2.53 )	0.82
Model I	1	1.59 ( 0.57–4.40 )	1.00 ( 0.34–2.88 )	0.99
Model II	1	1.47 ( 0.47–4.58 )	0.80 ( 0.24–2.61 )	0.53
Model III	1	1.36 ( 0.49–3.72 )	0.88 ( 0.31–2.50 )	0.79

FPG, fasting plasma glucose; HDL, high-density lipoprotein; TG, triacylglycerol; WC waist circumference; LDL, low-density lipoprotein ^a^ *P* for trend result from Mantel-Haenszelextention test; ^b^Adjusted for age; ^c^Adjusted for age, BMI (other than overweight, obesity and abdominal obesity), smoking, physical activity, taking estrogen tablets, familial history of diabetes and stroke; ^d^Adjusted for age, BMI (other than overweight, obesity and abdominal obesity), smoking, physical activity, taking estrogen tablets, familial history of diabetes, stroke and energy intake.

## Discussion

Based on previous observational studies, the relationship between carbohydrate intake, obesity, and markers of cardiovascular disease remain unclear. This was the first study in Iranian women that investigated how low-CHO diet scores relate to overweight, obesity, and markers of cardiovascular disease. The findings of this cross-sectional study showed that there was no significant relationship between low-CHO diet score with overweight, obesity and cardiovascular risk factors among this group of women.

In the present study, women in the highest tertile of low-carbohydrate diet consumed a smaller percentage of their diet as carbohydrates as well as a smaller amount of whole grains, refined grains and fruits (similar to the study by Shirani et al)^10^ and a greater amount of meat, variety of fats (cholesterol, SFA, MUFA and PUFA) and dairy products. However, vegetable intake did not differ among the tertiles, which is similar to previous work by Eslamian et al.^29^ Importantly, the lowest carbohydrate intake tertile (tertile 3) still had an average carbohydrate intake (53% of total energy intake) above the definition of a low carbohydrate diet (<45% of energy from carbohydrates).^11^ Thus, if a low carbohydrate diet did relate to anthropometric or cardiovascular disease outcomes, it is unlikely that this relationship would be seen in the present group of women.

This study found no significant relationship between low-carbohydrate-diet score and odds ratio of high triglycerides, high systolic and diastolic blood pressure and low HDL cholesterol. Previous work investigating these relationships in other adult populations has shown mixed results. A recent systematic review and meta-analysis of cohort studies showed that the highest score of the low-carbohydrate diet was marginally associated with the risk of diabetes mellitus.^30^ However, both cross-sectional and retrospective cohort studies have also highlighted that diets low in carbohydrate and high in protein and fat were not associated with an increased risk of coronary heart disease in women.^10,13,16,29^ In the current study, beyond the above-explained lack of a tertile with a carbohydrate intake < 45% of total energy, there are multiple potential contributors to the null results. These could include fatty acid intake and total dietary glycemic index. Previous epidemiological studies have demonstrated that saturated fatty acids and trans fatty acids, but not total dietary fat was associated with an increased risk of coronary heart disease whilst polyunsaturated and monounsaturated fats associated with reduced risk of this disease.^31^ In the present study, individuals in the highest tertile of the low-carbohydrate-diet score had higher intakes of saturated fat, as well as higher intakes of MUFA and PUFA, possibly contributing to null results. Also, total daily GI was significantly different among the tertiles of the low-CHO diet score. Previous cohort studies in adults,^32-34^ have shown that GI score was positively associated with the risk of non- insulin-dependent diabetes and inversely associated with HDL cholesterol. In the present study, the magnitude of difference in GI among tertiles was small. This may help to further explain why low-CHO diet scores did not relate to outcome measures.

Overweight, obesity and waist circumference did not relate to low-CHO diet scores. Previous cohort studies and randomized trials have shown mixed results as to the relationship between weight and low-carbohydrate diets.^35-40^ A systematic review of randomized trials highlighted that a low-CHO diet may improve weight status over 1 year, but longer term, this relationship was unclear.^41^ A more recent meta-analysis of longer-term cohort studies showed that carbohydrate intake did not relate to the risk of obesity.^42^ However, this review did state many important limitations including inconsistent reporting of potential confounders and non-standardized classification of dietary intakes. In the current study, it is possible the lack of relationship between body weight and low-CHO diet scores was affected by most women having a normal BMI and waist circumference. Further, energy intake was similar among tertiles. Although total daily GI differed among tertiles, it is likely that this does not relate to weight status as a previous meta-analysis has shown that long term low GI or low glycemic load diets did not beneficially affect fat mass in adults.^43^

Although this study had multiple strengths, there were some limitations. First, the cause and effect relationship is unknown because of the cross-sectional nature of the study. As previously described, the average carbohydrate intake of lowest carbohydrate tertile (tertile 3) was 53% of total energy intake, above the definition of low-CHO diet (<45% of total energy intake). Thus, without a tertile on a low-CHO diet, it may limit the ability to identify potential differences in dependent measures based on carbohydrate intake. Further, although the food frequency questionnaire used was validated, self-reported dietary intakes have the possibility of under-reporting or over-reporting dietary intakes.^44^ For outcomes related to weight and waist circumference, since the majority of women were classified as normal weight, it is unclear if the sample size was sufficient to investigate obesity-related outcomes. Also, because body composition was not assessed, it is unclear how body fat percentage relates to low-CHO diet scores. Lastly, due to sample size, the results of this study are likely not generalizable to the general population of women in Iran.

## Conclusion

Diets that were low in carbohydrates were not significantly associated with overweight, obesity and cardiovascular risk factors in Iranian women. Due to the cross-sectional design of the study, causality cannot be determined. Therefore, further well-designed studies, such as randomized trials, are required to confirm these results.

## Competing interests

None.

## Ethical approval

This study was approved by the ethical committee and research council of Tehran University of Medical Sciences (number 9411323006).

## Supplementary Materials


Supplementary file 1 contains the STROBE checklist.
